# Transient Expression of IL-17A in Foxp3 Fate-Tracked Cells in *Porphyromonas gingivalis*-Mediated Oral Dysbiosis

**DOI:** 10.3389/fimmu.2020.00677

**Published:** 2020-04-23

**Authors:** Peter D. Bittner-Eddy, Lori A. Fischer, Massimo Costalonga

**Affiliations:** Division of Periodontology, Department of Developmental and Surgical Sciences, School of Dentistry, University of Minnesota, Minneapolis, MN, United States

**Keywords:** Foxp3, Treg cells, Th17 cells, fate-tracking, IL-17A, periodontitis, *Porphyromonas gingivalis*

## Abstract

In periodontitis *Porphyromonas gingivalis* contributes to the development of a dysbiotic oral microbiome. This altered ecosystem elicits a diverse innate and adaptive immune response that simultaneously involves Th1, Th17, and Treg cells. It has been shown that Th17 cells can alter their gene expression to produce interferon-gamma (IFN-γ). Forkhead box P3 (Foxp3) is considered the master regulator of Treg cells that produce inhibitory cytokines like IL-10. Differentiation pathways that lead to Th17 and Treg cells from naïve progenitors are considered antagonistic. However, it has been reported that Treg cells expressing IL-17A as well as IFN-γ producing Th17 cells have been observed in several inflammatory conditions. Each scenario appears plausible with T cell transdifferentiation resulting from persistent microbial challenge and consequent inflammation. We established that oral colonization with *P. gingivalis* drives an initial IL-17A dominated Th17 response in the oral mucosa that is dependent on intraepithelial Langerhans cells (LCs). We hypothesized that Treg cells contribute to this initial IL-17A response through transient expression of IL-17A and that persistent mucosal colonization with *P. gingivalis* drives Th17 cells toward an IFN-γ phenotype at later stages of infection. We utilized fate-tracking mice where IL-17A- or Foxp3-promoter activity drives the permanent expression of red fluorescent protein tdTomato to test our hypothesis. At day 28 of infection timeline, Th17 cells dominated in the oral mucosa, outnumbering Th1 cells by 3:1. By day 48 this dominance was inverted with Th1 cells outnumbering Th17 cells by nearly 2:1. Tracking tdTomato^+^ Th17 cells revealed only sporadic transdifferentiation to an IFN-γ-producing phenotype by day 48; the appearance of Th1 cells at day 48 was due to a late *de novo* Th1 response. tdTomato^+^ Foxp3^+^ T cells were 35% of the total live CD4^+^T cells in the oral mucosa and 3.9% of them developed a transient IL-17A-producing phenotype by day 28. Interestingly, by day 48 these IL-17A-producing Foxp3^+^ T cells had disappeared. Therefore, persistent oral *P. gingivalis* infection stimulates an initial IL-17A-biased response led by Th17 cells and a small but significant number of IL-17A-expressing Treg cells that changes into a late *de novo* Th1 response with only sporadic transdifferentiation of Th17 cells.

## Introduction

Periodontitis is a destructive inflammatory disease that leads to progressive destruction of the soft tissues and alveolar bone supporting the tooth. This disease represents the sixth most prevalent disease worldwide (1). Severe periodontitis affects between 8 and 10% of the adult population in western and developing countries (2, 3). Periodontitis is associated with persistent colonization of the periodontal pocket by a consortia of microorganisms organized as a multispecies biofilm that contains symbionts, pathobionts and keystone bacterial pathogens like *Porphyromonas gingivalis* (*Pg*) (4). Virulence factors of microorganisms like *Pg* induce inflammation thereby altering the nutrient foundation of the microbial community resulting in population shifts within the consortia (5). Although poorly pathogenic in mono-colonized germ free mice, the dysbiosis induced by *Pg* in specific pathogen free mice (6) elicits an adaptive CD4^+^ T cell response against a wide spectrum of antigens originating from the expanded pathobiont population. The resulting immune response eventually leads to progressive destruction of the soft connective tissues and alveolar bone holding teeth in place (7). Understanding the immunopathogenesis of periodontitis is critical to strategies that seek to prevent, treat or predict future occurrence of disease.

We address the immunopathogenesis of periodontitis by determining how the innate and adaptive immune response behaves against new microbial threats entering the oral ecosystem. Here, activated CD4^+^ T and B cells are key players in modulating homeostasis of the bone supporting the tooth following the microbial insult (8–14) and reviewed in (5). CD4^+^ T helper (Th) 1, Th17 and T regulatory cells (Treg) often coexist in the same periodontal lesion. We currently do not know if these CD4^+^ T cells are generated and maintained as independent lineages or whether in the face of persistent dysbiosis and a chronic disease state they exhibit phenotypic plasticity and shift over time to different pathogenic potentials.

Situated proximal to the mucosal microbial biofilm in the periodontal pocket, epithelial and Langerhans cells (LCs) sample the microbial environment, recruit the subepithelial inflammatory infiltrate and modulate the adaptive response. We have established that Th17 differentiation of *Pg*-specific naïve CD4^+^ T cells *in vivo* is sustained by LCs (15). Current research suggests that in periodontitis Th17 cells and their signature cytokine, IL-17A, are central to bone destruction by promoting osteoclastogenesis (16–18). Although other evidence suggests that IL-17A can be protective (19), many suggest that IFN-γ-producing Th1 cells also drive alveolar bone destruction (8, 12, 20). Plasticity of Th17 cells is well documented (21–24), and a late developmental switch to IFN-γ expression in Th17 cells has been implicated in the pathologies of a number of inflammatory autoimmune diseases (25–28).

T regulatory cells (Treg) regulate the activity of T cells of several different phenotypes. The nuclear protein Forkhead box P3 (Foxp3) is considered the master regulator of Treg cells. However, the notion of Foxp3-expressing cells as a stable lineage of terminally differentiated Treg cells is controversial. Treg cells generally expressing IL-10 can also switch to IFN-γ-producing Th1-like cells (29) and even IL-17A-producing Th17-like cells (30) under certain inflammatory conditions [reviewed in (31–33)]. Currently, Treg cells are proposed as a heterogeneous pool, and while the majority of them are lineage stable, a minor uncommitted population does retain the capacity of reprogramming to a different phenotype [reviewed in (34)]. Although some evidence is present in humans (35), *in vivo* mouse models of inflammatory colitis provide the strongest evidence of Treg to Th17 reprogramming. In a murine model of inflammatory colitis CCR6^+^ Tregs producing retinoic acid orphan receptor (ROR) γt apparently drive the inflammation of the large intestine. In this model, CCL20 inhibits Foxp3 expression and directs former Tregs toward IL-17A expression. Analysis of peripheral blood from patients with ulcerative colitis suggests a similar process could occur in humans (35). Finally, IFNγ^+^ Th1/Tregs have been described in a murine model of atherosclerosis, suggesting Treg-Th1 plasticity could also occur (36).

What is unknown in periodontitis is whether differentiated CD4^+^ T cells modulate their response by re-programming cytokine expression when encountering persistent dysbiosis and heightened ability of pathobionts to cross the oral mucosal barrier. Here we tested the hypothesis that persistent *Pg* colonization creates the conditions to drive Treg and Th17 transdifferentiation. To test this hypothesis, we utilized Th17 and Treg lineage-tracing mice (23, 29) orally inoculated with *Pg* at 4-day intervals to mimic persistent dysbiosis. CD4^+^ T cells permanently labeled with a fluorescent reporter protein after activation of IL-17A or Foxp3 promoters were tested for expression of unorthodox cytokines. In this manuscript we present the relative proportion of Th17, Th17-derived Th1-like cells expressing IFN-γ, new Th1 cells, Treg and Treg-derived Th17-like that express IL-17A in murine oral mucosal and cervical lymph nodes over time after persistent oral colonization with *Pg*.

## Materials and Methods

### Animals

All animal experiments were reviewed and approved by the Institutional Animal Care and Use Committee of the University of Minnesota, and performed on age and sex-matched (8 to 10 weeks) mice or littermates, where appropriate. All mice were housed in microisolator cages with food and water *ad libitum* in a specific pathogen free animal facility. C57BL/6J mice were originally obtained from Jackson Laboratories (Bar Harbor, ME, United States). Experimental IL-17A^cre^ fate-tracking mice were bred by us as previously described (37). Experimental tamoxifen-inducible Treg-fate-tracking animals (Foxp3^cre^ fate-tracking mice) were generated by crossing B6.Cg-Gt(ROSA)26Stm14(CAG-tdTomato)Hze/J (Jackson #007914) animals with Foxp3tm9(EGFP/cre/ERT2)Ayr/J animals [Jackson #016961, (38)] to generate F_1_ hybrids. F_1_ animals were then cross bred to generate F_2_ breeders and experimental animals. F_2_ experimental animals were Rosa26-tdTomato homozygous and either Foxp3-cre-ERT2 hemizygous (males) or Foxp3-cre-ERT2 homozygous (females). In these animals, removal of the floxed stop codon and expression of tdTomato red fluorescent protein is tightly regulated by administration of tamoxifen. Inducible activation of Foxp3-cre is critical here as Foxp3 is active developmentally, and stochastic expression of Foxp3-cre can lead to mice with non-specific expression of tdTomato in multiple cell lineages (37).

### Administration of *P. gingivalis*

All mice were treated for 10 days with Sulfamethoxazole/Trimethoprim (SMZ-TMP), with a 4-day pause without antibiotics before administration of *Pg* or PBS as previously described (39, 40). The SMZ-TMP treatment is solely used to create an ecological niche in the oral microflora of mice enabling *Pg* colonization, and has no significant lasting or other effects. *Pg* strain ATCC 53977 (10^9^ CFU/mouse) or PBS was administered in 2% carboxymethylcellulose via atraumatic oral gavage with a ball-tipped needle as previously described every 4 days until completion of the experiment (39, 40) to ensure persistent *Pg*-induced dysbiosis (6).

### Administration of Tamoxifen

Tamoxifen (Millipore-Sigma, St Louis, MO, United States) was prepared at 40 mg/ml in olive oil with 10% (v/v) ethanol. 200 μl of tamoxifen stock solution was given per mouse via oral gavage on days 14, 15, and 17 of the experimental timeline (38). Tamoxifen was administered as a brief “pulse” after *Pg* inoculation at day 14 for two specific reasons. First, we needed to strike a balance between having a sufficient number of newly generated antigen-specific Foxp3 Tregs present in the mouse and allowing sufficient time for these now tdTomato-expressing cells to respond to dysbiotic changes induced by persistent *Pg*. Second, we want to limit the labeling of conventional CD4 T cells that may transiently express Foxp3 during early lineage commitment (41). Additionally, this system also avoids mislabeling of tissues due to stochastic activation of Foxp3 that we reported to occur during embryogenesis (37).

### Identification of *P. gingivalis*-Specific Antigen-Experienced CD4^+^ T Cells in Cervical Lymph Nodes by Flow Cytometry

Single-cell suspensions were isolated by standard techniques from cervical lymph nodes of C57BL/6J mice following sustained *Pg* inoculation or PBS (sham) treatment. Cells were stained with viability dye Zombie Aqua (BioLegend, San Diego, CA, United States) and Fc receptors blocked using anti CD16/CD32 antibody (eBioscience; clone 93). Cells were stained using a gingipain-specific MHC class II tetramer (pR/Kgp:I-A^b^) as previously described (42) and then with anti-mouse CD3 (BioLegend; clone 17A2), CD4 (eBioscience; clone RM4-5), CD8α (eBioscience; clone 53-6.7), CD44 (BioLegend; clone IM7) and B220 (eBioscience; clone RA3-6B2) fluorochrome-conjugated mAbs to distinguish antigen-experienced CD4^+^ T cells from B cells, CD8^+^ T cells and naïve lymphocytes. Cells were sorted on an LSR II flow cytometer (BD Biosciences, San Jose, CA, United States) and fluorescence emissions analyzed with FlowJo software (v10.4.1;Tree Star, Ashland, OR, United States).

### Analysis of Immune Cells From Oral Mucosa by Flow Cytometry

Mice were given an intravenous injection of 1.25 μg FITC-conjugated rat anti-mouse CD45 monoclonal antibody (eBioscience San Diego, CA, United States; clone 30-F11) as described previously to exclude blood resident immune cells from subsequent analyses (43). Oral mucosa (maxillary and mandibular gingiva, buccal, and posterior hard-palate tissues) was harvested and single-cell suspensions generated as described (43). Cells were stained with Zombie Aqua, Fc receptors blocked with anti-mouse CD16/CD32 antibody followed by incubation with a panel of mAbs that included anti-mouse CD45 mAb conjugated to PE (eBioscience; clone 30-F11), anti-mouse CD3, CD4, CD8α, TCR γδ(BioLegend; clone GL3), and TCR β (BioLegend; clone H57-597) fluorochrome-conjugated mAbs. Cells were acquired on an LSR II flow cytometer and fluorescence emissions analyzed with FlowJo software (v10.4.1;Tree Star, Ashland, OR, United States).

### Identification of Cytokines and Foxp3 in CD4^+^ T Cells Isolated From Oral Mucosa and Cervical Lymph Nodes

Single-cell suspensions from oral mucosal samples or cervical lymph nodes were harvested and cultured overnight in complete EHAA (Irvine Scientific, Santa Ana, CA, United States) supplemented with 2.5 μg/ml of PHA-L (Millipore-Sigma). Cells were polyclonally stimulated with PMA-Ionomycin in the presence of brefeldin A as previously described (42). After 6 h of stimulation, cells were incubated with Zombie Aqua, anti-mouse CD16/CD32 antibody and surface stained with the panel of mAbs described above. Cells obtained from oral mucosal tissues were not additionally stained using the gingipain-displaying MHC class II tetramer (pR/Kgp:I-A^b^) (42) as insufficient gingipain-specific CD4^+^ T cells (*Pg*-specific) are present in this tissue type for robust analysis. For cells obtained from IL-17A^cre^ fate-tracking and Foxp3^cre^ fate-tracking mice, fixation was done in 1:1 ratio of 4% paraformaldehyde and BD Perm/Wash (Cat. #51-2091KZ; BD Biosciences, San Jose, CA, United States) to preserve tdTomato and GFP signals during permeabilization. Permeablized cells were then stained with IL-17A (eBioscience; clone eBio17B7), IFN-γ (Biolegend; clone XMG1.2), or IL-10 (Biolegend; clone JES5-16E3) fluorochrome-conjugated mAbs. Cells from Foxp3^cre^ fate-tracking mice were incubated with anti-mouse Foxp3:AF488 mAb (Biolegend; clone MF-14) concurrently with cytokine mAbs. Cells were acquired on an LSR II flow cytometer and fluorescence emissions analyzed with FlowJo software (v10.4.1;Tree Star, Ashland, OR, United States).

### Statistical Analysis

Data was analyzed and plotted using Prism 7 software (GraphPad Software, San Diego, CA, United States) and displayed as means ± SEM. Each data point is from at least three independent experiments. Means between two groups were compared using Student’s *t*-test and between multiple groups with one-way ANOVA with *post hoc* analyses for multiple comparisons. *P*-values of 0.05 or less were considered significant. Unpaired or paired *t-*tests were performed as dictated by the data set being analyzed. Non-parametric comparisons were assessed with Mann-Whitney *U* test.

Data for the MFI of GFP was analyzed by fitting a mixed effects model with paired values (tdTomato +ve vs. tdTomato −ve in the same sample), rather than by repeated-measures ANOVA. Repeated measure ANOVA is unable to process missing values, which occurred in mice where tdTomato^+^ IL-17A^+^ or tdTomato^+^ IFN-γ^+^ populations were not detected. A *post hoc* Sidak’s multiple comparisons test was utilized to determine *p*-values of comparisons found significant by the initial mixed-effects analysis.

## Results

### IL-17A-Expressing *Pg*-Specific CD4^+^ T Cells in Cervical Lymph Nodes Peak at Day 28 While IFN-γ Expressing Cells Increase From Day 28 to 56

**FIGURE 1:**
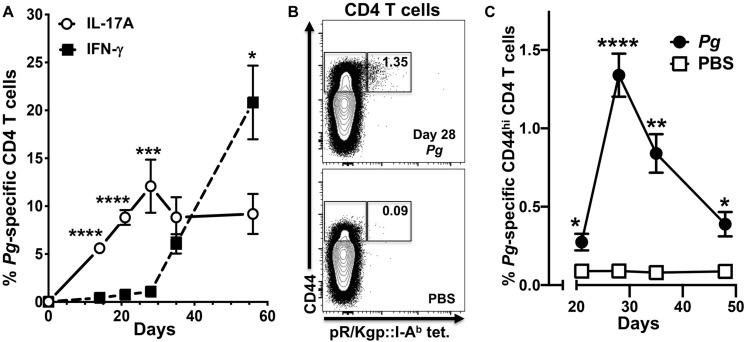
The percentage of *P. gingivalis*-specific CD4^+^ T cells expressing IFN-γ increases from day 28 in cervical lymph nodes. Single-cell suspensions from cervical lymph nodes of C57BL/6J mice orally inoculated with *Pg* or PBS were enriched for CD4^+^ cells at defined time points and stimulated with PMA/ionomycin in the presence of brefeldin A. Cells were surface stained with anti-mouse CD3, B220, CD8α, CD4, CD44 fluorochrome-conjugated mAbs and pR/Kgp:I-A^b^ tetramer and then intracellularly with anti-mouse IL-17A and IFN-γ mAbs to identify antigen-experienced gingipain-specific CD4^+^ T cells by flow cytometry (gated as CD44^bright^ CD3^+^ CD4^+^ pR/Kgp-IA^b+^ B220^–^ CD8α^–^). Data was pooled from three independent experiments totaling at least 8 mice per group and displayed as mean percentage ± SEM. **(A)** Summary data of total antigen-experienced pR/Kgp:I-A^b^ tetramer positive CD4^+^ T cells that expressed either IL-17A or IFN-γ. **(B)** Representative FACS plots from a *Pg* and PBS mouse showing pR/Kgp:I-A^b^ tetramer positive CD4^+^ T cells. Gates are drawn around antigen-experienced CD4^+^ T cells identified as CD44^bright^. The frequency of pR/Kgp:I-A^b^ tetramer positive cells identified as a percentage of the total antigen-experienced CD4^+^ T cell population is given. **(C)** Summary data of percentage of antigen-experienced pR/Kgp:I-A^b^ tetramer positive CD4^+^ T cells. Percentages were compared using two-tailed Student’s *t*-test. *p < 0.05, **p < 0.01, ***p < 0.001, ****p < 0.0001.

We have shown previously that Th17 cells are predominant in the early adaptive CD4^+^ T cell response to *Pg* in an oral model of periodontitis, and that this response is critically dependent on LCs (39). To study the dynamics of an evolving adaptive response to *Pg*, we tracked expression of IL-17A and IFN-γ in *Pg*-specific antigen-experienced CD4^+^ T cells isolated from cervical lymph nodes (CLN) of *Pg* or PBS-treated C57BL/6J mice over the course of 56 days. The *Pg*-specific Th17 response increased and peaked at day 28, and was significantly greater than the minor Th1 response we observed at days 14–28 (Figure 1A). While the *Pg*-specific Th17 response plateaued from day 28 to 56, the frequency of *Pg*-specific Th1 cells increased substantially by day 35, and at the experimental endpoint (day 56) it was significantly higher than the frequency of *Pg*-specific Th17 cells (Figure 1A). The frequency of antigen-experienced *Pg*-specific CD4^+^ T cells in CLN as a percentage of total CD44^hi^ CD4^+^ T cells (Figure 1B) peaked at day 28 and then declined but was still significantly higher than that observed in PBS treated mice at all time points examined (Figure 1C).

### Total CD4^+^ T Cell Response Against Oral *Pg* in the Oral Mucosa Evolves From an Initial Th17 Response to One Dominated by Th1

**FIGURE 2:**
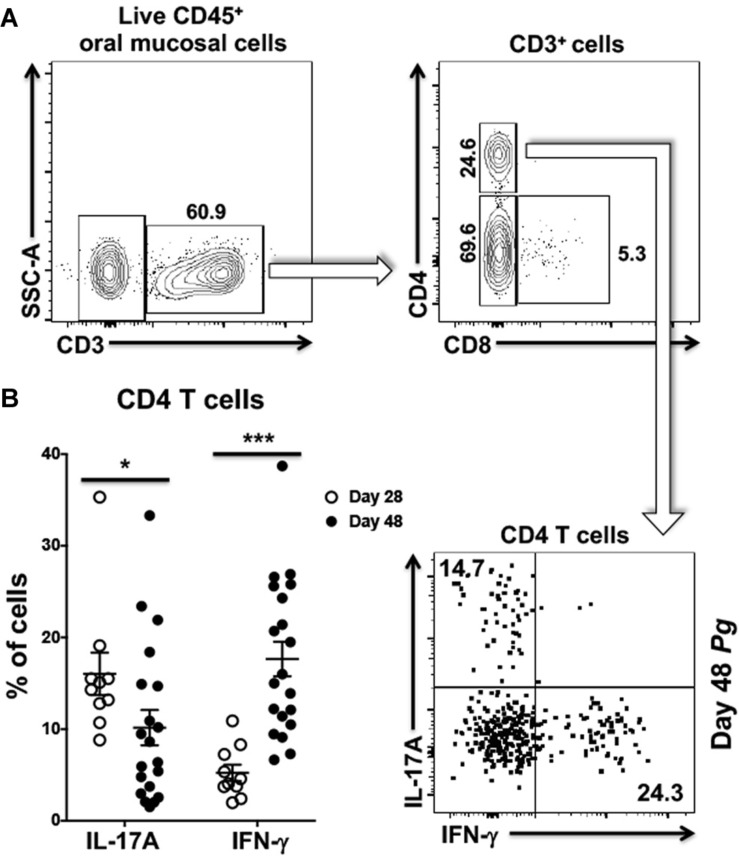
Frequency of Th1 cells significantly increases within the oral mucosa of *P. gingivalis* inoculated mice by day 48. **(A)** Representative flow cytometry plots showing the gating strategy used to identify Th1 (IFN-γ^+^) and Th17 (IL-17A^+^) cells within the oral mucosa. Numbers alongside gates indicate% of cells within that gate. **(B)** Summary data showing individual data points from three experiments examining the frequency of Th1 and Th17 recovered from the oral mucosa of mice at day 28 and day 48. Means ± SEM are plotted. Groups were compared with two-tailed Student’s *t*-test. *p < 0.05, ***p < 0.001.

We next examined CD4^+^ T cells recovered from the oral mucosa of C57BL/6J mice for expression of IFN-γ and IL-17A (Figure 2A). Here we chose to examine day 28 and day 48 based on changes we observed occurring in cells analyzed from cervical lymph nodes of *Pg* inoculated mice. In this analysis we found significantly more IL-17A expressing CD4^+^ T cells at day 28 compared to day 48 and fewer cells expressing IFN-γ at day 28 compared to day 48 (Figure 2B). In contrast, the frequency of IL-17A expressing CD4^+^ T cells decreased significantly by day 48 with a significant increase in the frequency of CD4^+^ T cells expressing IFN-γ. At day 28 Th17 cells were dominant in the murine oral mucosa out numbering Th1 cells by more than 3:1, but by day 48 this dominance was inverted with Th1 cells now outnumbering Th17 cells by close to 2:1. This phenotype shift in the overall mucosal CD4^+^ T cell response is consistent with the observations in cervical lymph nodes we reported earlier (Figure 1).

### At Homeostasis tdTomato Expression Marks Three Distinct IL-17A^+^ T Cell Populations in the Oral Mucosa of IL-17A^cre^ Fate-Tracking Mice

**FIGURE 3:**
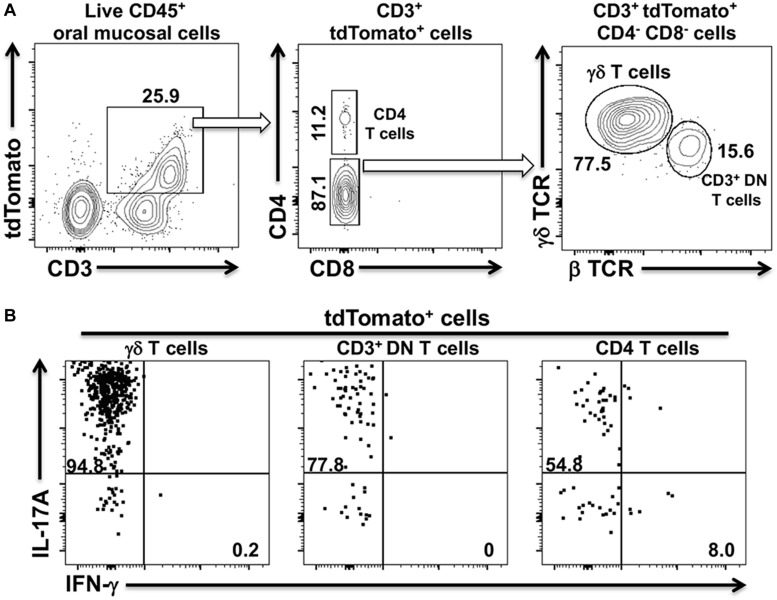
tdTomato expression in three distinct CD3^+^ T cells populations within the oral mucosa of IL-17A^cre^ fate-tracking mice. Oral mucosa was harvested from IL-17A^cre^ fate-tracking mice and single cell suspensions prepared from the tissue for subsequent analysis by flow cytometry. Single cell suspensions were stained with vitality dye Zombie Aqua followed by a panel of anti-mouse mAbs to identify immune cell subsets expressing tdTomato. Cells were counted by flow cytometry and analyzed by FlowJo software. **(A)** Representative flow cytometry plots showing gating strategy to identify three tdTomato^+^ cell populations. Numbers indicate the% of cells within a particular gate. Live CD4^+^ T cells were identified as Zombie Aqua^lo^, CD45^+^, CD3^+^, CD4^+^, CD8α^–^, NK1.1^–^; live γδT cells as Zombie Aqua^lo^, CD45^+^, CD3^+^, γδ TCR^+^, β TCR^–^, CD4^–^, CD8α^–^, NK1.1^–^; live CD3^+^ DN T cells as Zombie Aqua^lo^, CD45^+^, CD3^+^, β TCR^+^, γδ TCR^–^, CD4^–^, CD8α^–^, NK1.1^–^. **(B)** Representative flow cytometry plots showing expression of IL-17A and IFN-γ in the three CD3^+^ tdTomato^+^ cell populations. Single cell suspensions obtained from oral mucosa were cultured and stimulated with PMA/ionomycin in the presence of brefeldin A. Cells were surface stained with anti-mouse mAbs as in **(A)** and then intracellularly with anti-mouse IL-17A and IFN-γ mAbs.

The shift from a Th17 to a Th1-type response in cervical lymph nodes and in the oral mucosa suggested that a dynamic remodeling of the adaptive CD4^+^ T cell response to *Pg-*induced dysbiosis can occur over time. To examine whether this remodeling might involve transdifferentiation of Th17 cells to a Th1-like phenotype, we generated IL-17A^cre^ fate-tracking mice. IL-17A/Cre expression allows the de-repression of a tdTomato red fluorescent reporter protein thereby permanently labeling the entire progeny of all IL-17A-expressing cells (23, 37). In IL-17A^cre^ fate-tracking mice at homeostasis, we observed three distinct populations of CD3^+^ T cells in the oral mucosa that were marked by tdTomato expression (Figure 3A). These cells, therefore, must have had or currently have an active IL-17A promoter. Using this strategy, we identified significant numbers of conventional CD4^+^ T cells (Th17), γδ T cells and a population of CD3^+^ cells that have the β chain of the T cell receptor, but are marked as double-negative CD3^+^ T cells (CD3^+^ DN) due to the lack of CD4 and CD8 cell surface markers. This latter cell population may represent so-called mucosal-associated invariant T (MAIT) cells. Although we did not examine CD103 or CD69 expression in these CD3^+^ DN, NKT cells can be ruled out since they were negative for the diagnostic NK1.1 cell surface marker (data not shown). Notably, we did not observe tdTomato expressing CD8 T cells, also known as Tc17 cells. Permanent tdTomato labeling of cells that expressed IL-17A at any one time can reveal phenotypically plastic subpopulations that initiate the expression of unorthodox cytokines, like IFN-γ. At homeostasis, the three tdTomato^+^ CD3 T cells populations primarily expressed IL-17A with no evidence of plasticity toward a Th1-type phenotype (Figure 3B).

### Sporadic IFN-γ Expression by IL-17A^cre^-tdTomato^+^ T Helper Cells in the Oral Mucosa of *Pg* Inoculated Mice by Day 48

**FIGURE 4:**
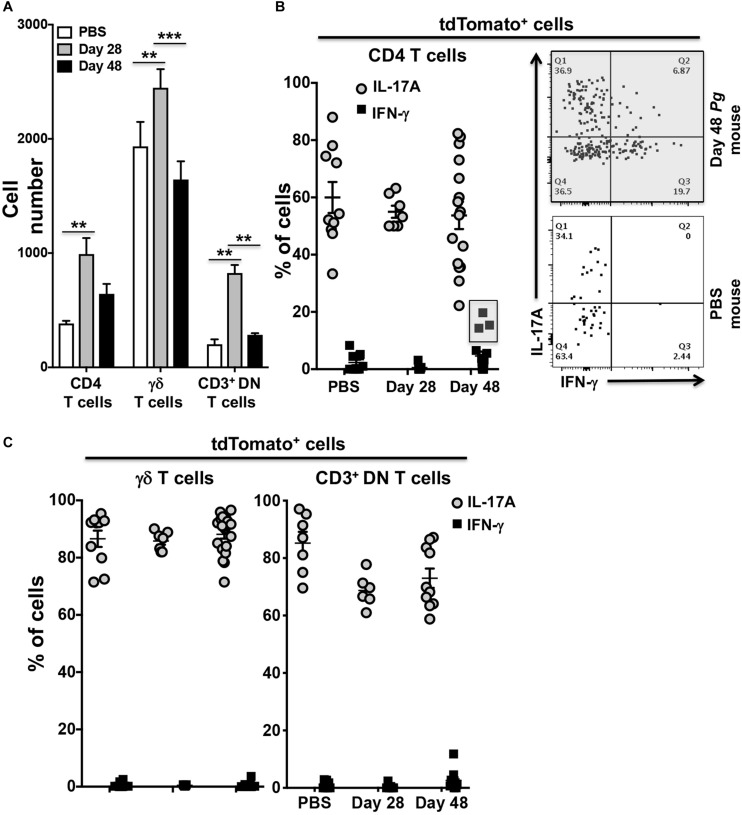
Expression of IFN-γ by tdTomato^+^ CD4^+^ T cells present within the oral mucosa of *P. gingivalis* inoculated mice is sporadic. IL-17A^cre^ fate-tracking mice were pre-treated with SMZ and then orally inoculated with either *P. gingivalis* (4 × 10^9^ cfu per ml) or vehicle (PBS). At day 28 or 48 oral mucosa was harvested and single-cell suspensions from single mice treated and stained with mAbs as described in Figure 3. **(A)** Summary data of total numbers of CD4^+^ T cells, CD3^+^ DN T cells and γδ T cells found in the oral mucosa of mice after 28 or 48 days. Cell types were identified from single cell suspensions as described in Figure 3. Cell numbers were normalized to 100,000 live non-immune cells to account for potential cell loss during processing and counting. Data are from 3 experiments with at least 2 mice per time point and are plotted with means ± SEM. Means analyzed by two-tailed Student’s *t*-test. **p < 0.01, ***p < 0.001. **(B)** Summary data showing individual data points from three experiments examining the frequency of IL-17A and IFN-γ expression in IL-17A^cre^-tdTomato^+^ CD4^+^ T cells recovered from the oral mucosa of IL17A fate-tracking mice. Representative flow cytometry plots from a single PBS and *P. gingivalis* inoculated mouse at day 48 showing evidence of IFN-γ expression in IL-17A^cre^-tdTomato^+^ CD4^+^ T cells. **(C)** Summary data showing individual data points from three experiments examining the frequency of IL-17A and IFN- γ expression in IL-17A^cre^-tdTomato^+^ CD3^+^ DN T cells and γδ T cells recovered from the oral mucosa of IL-17A^cre^ fate-tracking mice.

To examine potential *Pg*-induced transdifferentiation in CD3^+^ T cells subsets, we analyzed cells isolated from the oral mucosa of IL-17A^cre^ fate-tracking mice at day 28 and 48 and compared them to PBS control mice. In an initial characterization of *Pg* infection, we found significant infiltration of CD4^+^ T cells, γδ T cells and CD3^+^ DN cells into the oral mucosa at day 28 compared to PBS controls (Figure 4A). γδ T cells were more abundant than CD4^+^ T cells or CD3^+^ DN cells, although the latter cell types showed greater increases relative to PBS control mice. All three populations, however, declined by day 48 relative to day 28 despite sustained *Pg* inoculation.

We next examined the IL-17A^cre^-tdTomato^+^ fraction amongst the CD4^+^ T cell, γδ T cell and CD3^+^ DN cell populations for expression of IL-17A and IFN-γ (Figures 4B,C). Expression of IFN-γ in these IL-17A^cre^-tdTomato^+^ cells would be evidence of phenotype plasticity. High frequencies of γδ T cells expressing IL-17A, averaging just under 90%, and very few γδ T cells expressing IFN-γ were observed (Figure 4C). IL-17A expression was more variable in CD4^+^ T cells, and to a lesser extent in CD3^+^ DN cells, across all three groups (Day 28, 48, and PBS), but showed no significant differences (Figures 4B,C). Although we only found a trend of increased frequency of IL-17A^cre^-tdTomato^+^ CD4^+^ T cells expressing IFN-γ at day 48 compared to day 28 (*p* = 0.07, Mann-Whitney *U* test), it is clear from three distinct outliers that Th17 plasticity can occur due to *Pg*-induced dysbiosis. In a sample size of 16 mice we found 3 mice that at day 48 had frequencies of IFN-γ expression in IL-17A-cre-tdTomato^+^ CD4^+^ T cells that exceeded 15% (Figure 4B – gray box). Moreover, in these 3 mice, IL-17A-cre-tdTomato^+^ CD4^+^ T cells that expressed both IFN-γ and IL-17A were also found (Figure 4B; upper right [Q2] flow cytometry dot-plot).

IL-17A^cre^ fate-tracking mice did not exhibit IL-17A^cre^-tdTomato^+^ neutrophils in the oral mucosa following inoculation with *Pg* (data not shown) indicating that neutrophils do not, and have not, expressed IL-17A at any one time in their ontogeny. Furthermore, there was no influx of IL-17A expressing NKT cells or Tc17 cells to the oral mucosa (44, 45).

### Fluorescent Foxp3^+^ Populations Generated in Foxp3^cre^ Fate-Tracking Mice

As we did not observe consistent transdifferentiation of Th17 cells to IFN-γ producing cells, we next sought to determine whether the CD4^+^ Treg response is reshaped after persistent exposure to oral *Pg*. Treg cells are a high frequency population in the murine oral mucosa producing anti-inflammatory cytokines like IL-10 at homeostasis. A shift toward production of pro-inflammatory cytokines such as IFN-γ and IL-17A could have significant implications for pathogenesis of periodontal disease.

**FIGURE 5:**
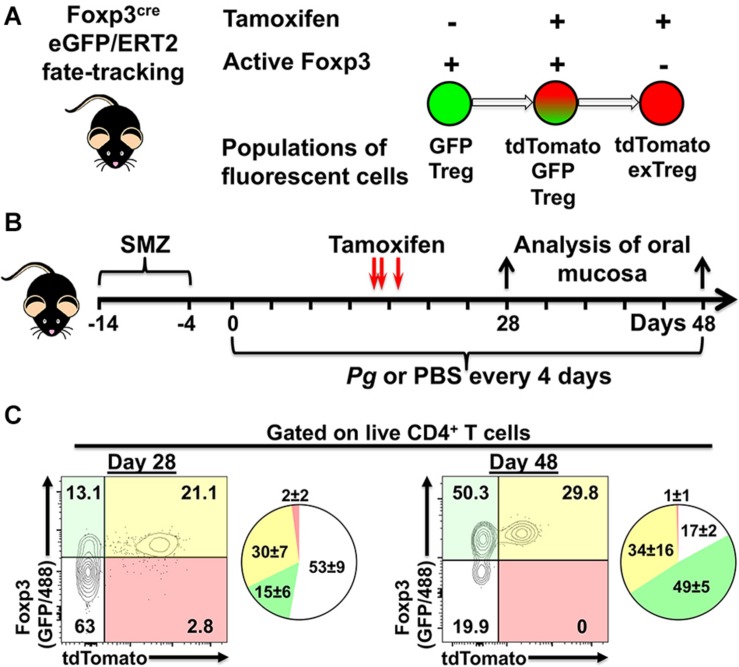
Experimental design to identify populations of fluorescently labeled Treg cells. **(A)** Potential populations of fluorescent Treg cells found in tamoxifen-treated Foxp3^cre^-GFP//ERT2 fate-tracking mice. **(B)** Experimental timeline for administration of *P. gingivalis* and, tamoxifen in experimental animals. **(C)** Representative flow cytometry plots gated on live CD4^+^ T cells from the oral mucosa of mice treated with tamoxifen and inoculated with *Pg* for 28 or 48 days. CD4^+^ T cells were identified as Zombie Aqua^lo^, CD45^+^, CD3^+^, CD4^+^, β TCR^+^, CD8α^–^. To augment GFP expressed from the Foxp3 promoter in these mice, cells were additionally stained with anti-mouse Foxp3 mAb conjugated to AF488. Numbers within the quadrants indicate% of the total CD4^+^ T cells gate. Summary pie charts show mean% ± SEM *N* = 4 mice.

Utilizing Foxp3^cre^ fate-tracking mice that track the fate of Foxp3-expressing Treg cells we have the potential to identify three populations of fluorescent cells in our experimental system due to tdTomato expression and GFP expression driven from the Foxp3 promoter (Figure 5A). The first population, single GFP^+^ cells (green fluorescent only) represents a population of Foxp3^+^ cells that differentiated only after tamoxifen has completely cleared from the host animal, so called late phase (tdTomato^–^) Treg cells (Figure 5A). Second, dual GFP^+^ (green fluorescent) and tdTomato^+^ (red fluorescent) cells, demonstrate currently active Foxp3 promoter driving expression of GFP and cre/ERT2-dependent tdTomato expression in the presence of tamoxifen (Figure 5A). Lastly, single tdTomato^+^ cells (red fluorescent) represent a population where the Foxp3 promoter was active at the time of tamoxifen administration but that is no longer active at day 28 or day 48 of timeline (Figure 5B).

We found that oral administration of tamoxifen on day 14, 15, and 17 generated a robust population of dual positive tdTomato^+^ GFP^+^ Treg cells in oral mucosal tissues at day 28 and day 48 (Figures 5C). Day 48 mice had higher frequencies of single GFP^+^ cells compared to day 28 mice (49 ± 5 versus 15 ± 6), consistent with a longer period of post-tamoxifen recruitment of Treg cells in these mice. Interestingly, the increased frequency of single GFP^+^ cells appeared to come at the expense of non-Treg CD4^+^ T cells (GFP^–^ and tdTomato^–^ population). In mice, active tamoxifen persists for 22 h (46), giving us the opportunity with repeated administrations to simultaneously identify and track phenotype plasticity in early (tdTomato^+^) and late phase (tdTomato^–^) Treg cells in our experimental system. Additionally, delivering tamoxifen after the initiation of the adaptive immune response to *Pg*-induced dysbiosis also helps limit tdTomato-labeling of naïve cells committed to a Th17 phenotype that may transiently express Foxp3 (41).

### Transient IL-17A Expression by tdTomato^+^ Foxp3-GFP^+^ T Cells in the Oral Mucosa of Foxp3^cre^ Fate-Tracking Mice

**FIGURE 6:**
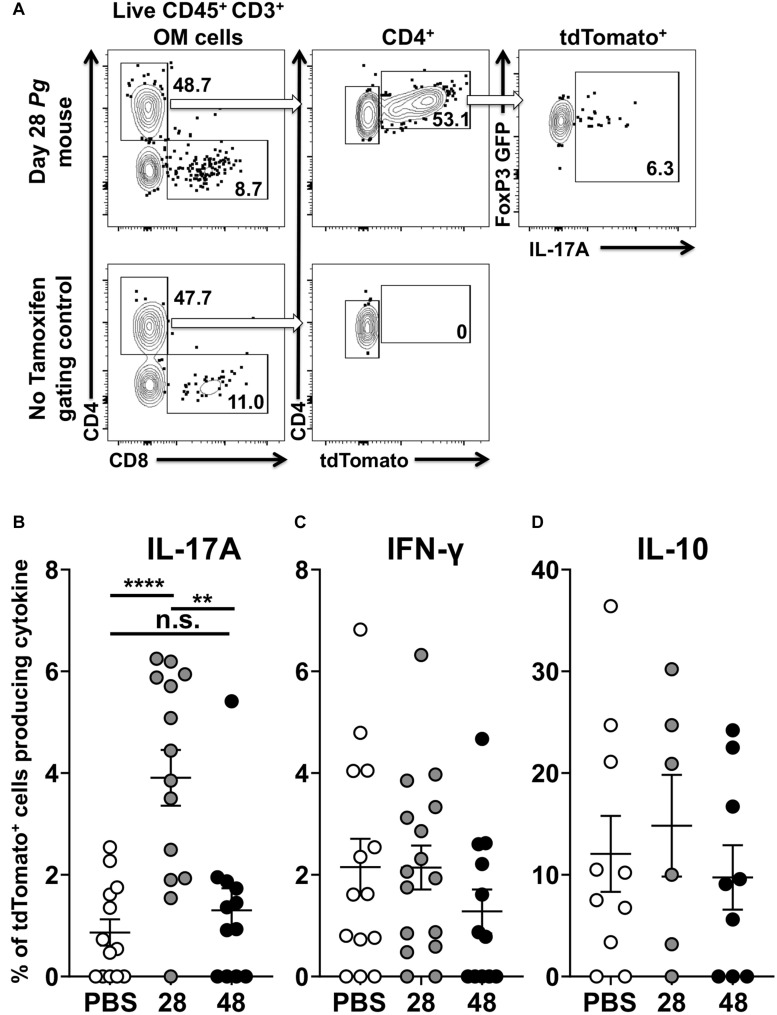
*P. gingivalis* induces transient IL-17A expression in Treg cells in the oral mucosa. Single cell suspensions were isolated from oral mucosa of *P. gingivalis* inoculated mice at 28 or 48 days. Single cell suspensions were cultured and stimulated with PMA/ionomycin in the presence of brefeldin A. Cultured cells were surface stained with anti-mouse CD3, B220, CD8α, and CD4 fluorochrome-conjugated mAbs to identify tdTomato^+^ CD4^+^ Treg cells by flow cytometry (gated as CD3^+^ CD4^+^ tdTomato^+^ B220^–^ CD8α^–^) and then intracellular stained with anti-mouse IL-17A, IFN-γ, and IL-10. **(A)** Representative flow cytometry plots showing gating strategy to identify the Foxp3^cre^-tdTomato^+^ cell population within the oral mucosa also expressing Foxp3-GFP and IL-17A. Numbers indicate the % of cells within the associated gate. **(B–D)** Means of the frequency of tdTomato^+^ CD4^+^ T cells expressing IL-17A **(B)**, IFN- γ **(C)**, or IL-10 **(D)** at day 28 (gray circle) and 48 (black circle) were compared to sham controls (white circle) using two-tailed Student’s *t*-test and presented as means ± SEM. **p < 0.01, ****p < 0.0001. Each circle represents two pooled oral mucosae from two mice.

Examining cytokine expression of single red and dual red/green fluorescent cells after the administration of tamoxifen elucidated the extent of transdifferentiation of Foxp3^+^ Treg cells (Figure 6A). Foxp3^cre^ fate-tracking mice not treated with tamoxifen, used as FACS gating controls, had either negligible numbers or frequently no tdTomato^+^ cells related to endogenous estrogen levels (Figure 6A). Next, we compared expression of IL-17A, IFN-γ, and IL-10 in tdTomato^+^ cells isolated from *Pg* and sham treated Foxp3^cre^-fate-tracking mice. Twenty eight days after initial *Pg* inoculation Foxp3^cre^-tdTomato^+^ T cells were 35% (range 22.7 to 47.6) of the CD4^+^ T cells we detect in the oral mucosa. At day 28, the Foxp3^cre^-tdTomato^+^ T cells that produced IL-17A were 3.9% (range 0.0 to 6.3). This was significantly higher than the 0.9% observed in sham-inoculated controls (*p* < 0.001). However, after 48 days of continuous *Pg* inoculation, the percentage of Foxp3^cre^-tdTomato^+^ T cells producing IL-17A had dropped significantly to 1.3% when compared to day 28 (*p* < 0.01). This frequency was not significantly different from sham-inoculated controls, suggesting here that by day 48 *Pg* is no longer sustaining IL-17A expression in fate-tracked Treg cells (Figure 6B). Interestingly, although we did identify Foxp3^cre^-tdTomato^+^ T cells expressing IFN-γ, the frequency was low and independent of *Pg* inoculation as there was no significant difference between sham- and *Pg-*inoculated mice at day 28 or 48 (Figure 6C). As expected we observed IL-10 expression in Foxp3^cre^-tdTomato^+^ T cells at homeostasis but their frequency did not increase after *Pg*-induced dysbiosis (Figure 6D).

### T Cells Producing IL-17A Maintain Expression of Foxp3 When Assessed by GFP Signal

**FIGURE 7:**
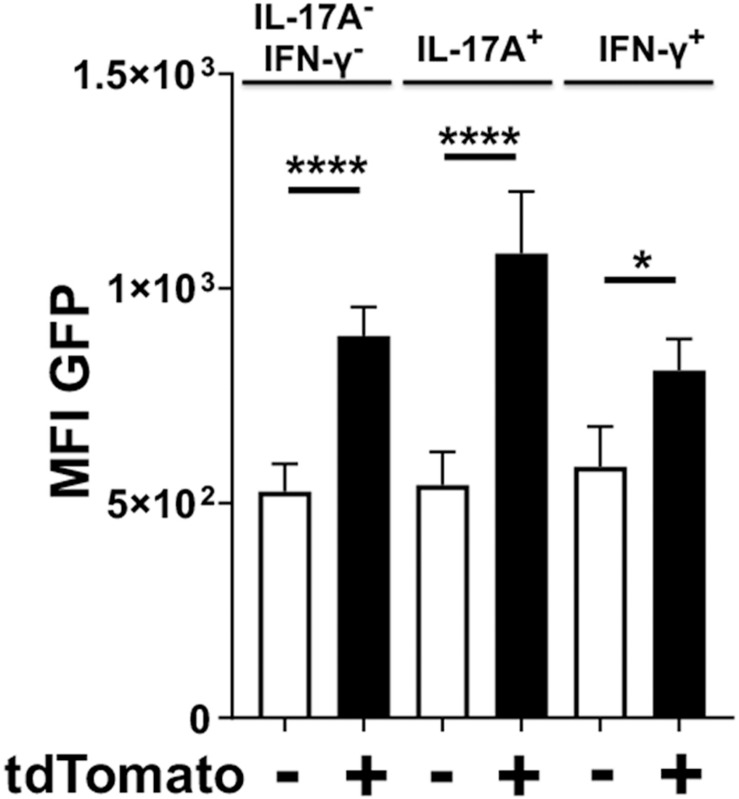
Foxp3^cre^-tdTomato^+^ Treg cells producing IL-17A have an active Foxp3 promoter. Foxp3^cre^-tdTomato^+^ CD4^+^ Treg cells from the oral mucosa were identified by flow cytometry (gated as CD3^+^ CD4^+^ tdTomato^+^ B220^–^ CD8α^–^). As the GFP signal in these mice was weak, anti-mouse FoxP3-Alexa488 was added during intracellular staining to increase the Foxp3 signal. The mean fluorescence intensity (MFI) of Foxp3-dependent green fluorescent protein (GFP) in populations of tdTomato^–^ (white bars) and tdTomato^+^ cells (black bars) from 20 mice are compared and presented for cells expressing IL-17A, IFN-γ or neither of the two cytokines. The GFP MFI was compared by fitting a mixed effects model with paired values. A *post hoc* Sidak’s multiple comparisons test was utilized to determine *p*-values of comparisons found significant by the initial mixed-effects analysis. *p < 0.05, ****p < 0.0001.

Whether the observed kinetics of IL-17A expression in Foxp3^cre^-tdTomato^+^ T cells was due to clonal contraction of transdifferentiated cells or reversion back to normal Treg phenotype was unclear. Therefore, we sought to determine whether these IL-17A producing Foxp3^cre^-tdTomato^+^ cells were simultaneously expressing Foxp3 or whether expression of IL-17A was paralleled by cessation of Foxp3 expression. While all cells with active Foxp3 promoters at the time of tamoxifen administration would be tdTomato^+^, only cells actively expressing Foxp3 at the time of analysis would be GFP^+^. In order to normalize the analysis across multiple experiments, a paired analysis was utilized to compare the mean fluorescence intensity (MFI) of the GFP signal between IL-17A- and IFNγ-producing cells that are tdTomato positive or negative (Figure 7). As expected, in the IL-17A^–^ IFN-γ^–^ populations, Foxp3^cre^-tdTomato^+^ T cells had a higher MFI of GFP than the tdTomato^–^ ones indicating an ongoing active Foxp3 promoter (Figure 7). Surprisingly though, in IL-17A-expressing populations the MFI of GFP was higher in Foxp3^cre^-tdTomato^+^ that in Foxp3^cre^-tdTomato^–^ T cells. Similarly, in the IFN-γ-expressing populations the GFP MFI was higher in Foxp3^cre^-tdTomato^+^ that in Foxp3^cre^-tdTomato^–^ T cells. This pattern indicates that the Foxp3 promoter continues to be active in IL-17A- and IFN-γ-expressing populations (Figure 7). In our experimental design, we used IL-17A mAbs conjugated to either APC or Brilliant Violet 421, so this result is not an artifact due to inadequate compensation of IL-17A signal spillover into the GFP or tdTomato channel. Lastly, Foxp3-GFP signal was still significantly higher in the Foxp3^cre^-tdTomato^+^ population than in the corresponding tdTomato^–^ population at day 48 (data not shown). This indicates that Foxp3 promoter activity remains stable in Foxp3^cre^-tdTomato^+^ cells for the duration of the experiment suggesting that Treg cells can shift to proinflammatory phenotypes while maintaining a transcription factor characteristic of regulatory T cells.

## Discussion

Our fate-tracking animal model with repeated inoculations of *Pg* accomplishes two objectives. First, it is representative of human periodontitis since the persistence of *Pg* is a characteristic of the disease. In humans, *Pg* is present at periodontitis sites in higher numbers than at healthy sites. *Pg* disappears below detectable levels after periodontal treatment and it reappears when disease returns and/or exacerbates (4, 47). Second, the adaptive response is best interpreted when the priming antigens are delivered synchronously. Since *Pg* is a keystone pathogen capable of inducing microbial dysbiosis in the oral cavity, the model allows the assessment of the dynamics of the immune response against *Pg* in the cervical LN as well as the local response against blooming pathobionts within the microflora over time. The phenotype of the adaptive response at these two locations is coherent.

### Transdifferentiation of IL-17A^cre^ Fate-Tracked Cells

It has been widely reported that diseases can lead to a changing microenvironment resulting in localized changes to the inflammatory cytokine milieu that reshapes the adaptive immune response (22–24, 48). With such changes, adaptive effector or tissue memory CD4^+^ T cell may be impacted through transdifferentiation leading to greater pathologic phenotypes (26, 49–52). We were interested in determining if the adaptive CD4^+^ T cell response to sustained dysbiosis elicited by the keystone pathogen *Pg* resulted in transdifferentiation in Th17 and Treg cells in the oral mucosa. To address this question, we used two different fate-tracking reporter mouse strains to examine plasticity in Th17 or Treg cells, in a murine model of periodontitis (23, 37). Transdifferentiation of Th17 cells is well documented (21–23, 26, 53) and a late developmental switch to IFN-γ expression in Th17 cells has been implicated in the pathologies of a diverse group of inflammatory autoimmune diseases such as psoriatic arthritis, Crohn’s disease, ulcerative colitis, type 1 diabetes, and multiple sclerosis (23, 26, 49–52, 54). Similarly, there have been reports that Treg cells also can transdifferentiate into IFN-γ-producing Th1-like cells (29, 30, 55–57).

Initially we found that *Pg*-specific CD4^+^ T cells identified in cervical lymph nodes that drain the oral mucosa switch phenotype from Th17 to a mix of Th17 and Th1 cells over a period of sustained oral colonization with *Pg*. The early Th17 response is consistent with what we have reported previously (39) and also in our analysis of CD4^+^ T cells isolated from NALT following oral inoculation with *Pg* (43). The late-stage switch to a Th1-type response seems a direct consequence of a *de novo* adaption against a persistent threat. Consistent with localized changes in the cytokine milieu, differentiated Th17 cells may re-program their cytokine expression when encountering a persistent pathogen across the oral mucosal barrier. Data from IL-17A^cre^ fate-tracking mouse indicates that a *de novo* response to persistent *Pg* occurs with Th1 cells dominating Th17 cells in the oral mucosa at late time points. Lack of consistent transdifferentiation in Th17 cells to express IFN-γ rules out sustained local environmental changes in oral mucosa driving changes in memory Th cells. *Pg*-induced dysbiosis may be the driver of this new Th1 response. *Pg* is considered a keystone pathogen so that continued exposure in the oral cavity to this bacterium may drive other pathobionts to become more numerous or more prone to intracellular survival like *Fusobacterium nucleatum* or *Aggregatibacter actinomycetemcomitans* (58–61). Pathobionts may induce an IFN-γ mediated-response to deal with this new intracellular threat. Persistent *Pg* inoculation and heightened inflammation may also result in bacteria invading further into the oral mucosa. Bypassing resident LCs in the epithelium may allow invading pathobionts to be phagocytosed by DCs located in the lamina propria. Consistent with this idea, it is interesting to note that in the absence of LCs, mice mount a robust Th1 response to *Pg* (39).

Notwithstanding differences in homing receptors that dictate tissue residency, we found that *Pg*-specific Th17 cells in mesenteric lymph nodes dominated the adaptive CD4^+^ T cell response even after persistent oral *Pg* presence (40). The contribution of recirculating memory Th1 cells potentially developed in the GI to the oral response is therefore unlikely. Moreover, the transition from an initial Th17 response to one dominated by infiltrating Th1 cells has also been reported for a number of other inflammatory diseases (23, 62–64). Interestingly, Harbour et al. report that, in addition to plasticity of Th17 cells driving inflammation in a mouse model of colitis, Th17 cells are also instrumental in driving pathogenic Th1 cells from naïve CD4^+^ T cell precursors (51).

In addition to a switch to a Th1 dominated response, we observed evidence of sporadic transdifferentiation of Th17 cells. Clear evidence of plasticity in these Th17 cells is reinforced by the detection of cells that co-express IL-17A and IFN-γ, which is a hallmark of transdifferentiated Th17 cells (23, 26, 51, 65). The difference of these outliers could be explained by the local cytokine environment and the relative levels of cytokines such as IL-23, IL-1β, IL-12, and TGF-β that have been implicated in either maintaining or driving local transdifferentiation in Th17 cells (22, 26, 53, 65).

γδ T cells that express IL-17A also have the capacity to exhibit plasticity and in some disease states have hallmarks of active histone modifications in genes that drive IFN-γ expression (66–69). Proinflammatory cytokines IL-1β and IL-23 have been reported to act in concert to induce IFN-γ expression in γδ T cells (67) and microRNAs have been shown to regulate IFN-γ plasticity in γδ T cells (68). In our analysis of γδ T cells we found no evidence of IFN-γ expression in the tdTomato^+^ γδ T fraction indicating that these cells do not exhibit plasticity in the oral mucosa following *Pg*-induced dysbiosis. Interestingly, of the 3 animals that did exhibit Th17 transdifferentiation to IFN-γ, we found no evidence of IFN-γ expression in the γδ T cells recovered from these same mice. This dichotomy suggests that there may be different signals driving transdifferentiation in mucosal Th17 and γδ T cell populations. Although IL-23 is important for maintaining Th17 cells, IL-23 in conjunction with IL-12 can drive transdifferentiation in Th17 cells by suppressing IL-17A expression and at the same time promoting IFN-γ expression through upregulation of T-bet (22, 23, 65, 70). Furthermore, pathogenic IFN-γ expression in transdifferentiated Th17 cells mediated by IL-23 is dependent on the basic-leucine zipper transcription factor, JunB (71, 72). Differential expression of JunB in Th17 cells and γδ T cells located in the oral mucosa of *Pg* inoculated mice may, therefore, account for our observation of lack of γδ T cell plasticity in the small number of mice where we observed it in Th17 cells.

In addition to Th17 and γδ T cells expressing IL-17A, we also identified a population of CD3^+^ TCRαβ^+^ T cells in the oral mucosa that were negative for CD4 and CD8 and expressed IL-17A. These so-called CD3^+^ double negative (DN) T cells have never been reported as a source of IL-17A in the oral mucosa. The number of CD3^+^ DN T cells increased in the oral mucosa in response to *Pg* but did not show evidence of IFN-γ expression. The origins of these CD3^+^ DN T cells remains somewhat controversial, but it is known that they are a heterogenous T cell population with capabilities to express both pro and anti-inflammatory cytokines in steady state and during inflammation (73). CD3^+^ DN T cells are typically found in low numbers in peripheral tissues but contributing IL-17A against viral and bacterial pathogens (74–76) and in autoimmune diseases such as psoriasis and Sjögren’s syndrome (77, 78). In the context of periodontal disease, we do not know if CD3^+^ DN T cells are contributing to host defense or exacerbating periodontitis by acting as an additional source of IL-17A. In a recent report, Sparber et al. described three lymphocyte sources of IL-17A in the murine tongue important for host defense against oropharyngeal candidiasis (79). One of these cell types, CD3^+^ TCRαβ^+^ T cells may include a population of CD3^+^ DN T cells, but were not defined further with CD4 and CD8 markers. The authors also did not examine the oral mucosa. Interestingly though, a significant contribution to resistance to oropharyngeal candidiasis were IL-17A-expressing innate lymphoid cells (ILC), characterized as CD3^–^, αβTCR^–^ and γδTCR^–^ (79). We did not observe ILC in our IL-17 fate-tracking mouse. This may reflect differences in residency of ILCs between the mouse tongue and oral mucosa (gingiva, buccal, and hard palate mucosa). Although this topic is subject to current debate (17, 80, 81), we also did not observe IL-17A^cre^-tdTomato^+^ neutrophils despite infection with *Pg* resulting in an influx of neutrophils. This suggests that, neutrophils are not a source IL-17A in our periodontitis model.

### Transdifferentiation of Foxp3^cre^ Fate-Tracked Cells

Tamoxifen administration induced permanent tdTomato labeling in cells with concurrent expression of Foxp3 and GFP. Naïve T cells in a TGF-β environment that will eventually commit to a Th17 lineage can co-express Foxp3 and RORγt (41). Therefore, we chose to initiate tamoxifen administration only after the majority of precursor cells capable of becoming Th17 in response to oral *Pg*-induced dysbiosis were activated and no longer transiently expressing Foxp3. *Pg*-induced dysbiosis leads to expression of IL-17A in Foxp3^cre^-tdTomato^+^ Treg cells after 28 days of oral *Pg* persistence. Strikingly, IL-17A expression was transient since the frequency of IL-17A expressing Foxp3^cre^-tdTomato^+^ Treg cells was significantly reduced after 48 days of persistent oral *Pg* colonization. We did not observe significant IL-17A plasticity of Tregs in sham-treated mice (PBS mice), indicating that microbial dysbiosis is necessary to drive this transient transdifferentiation process. Potentially some newly differentiated Th17 cells transiently expressing Foxp3 could have been misidentified as transdifferentiated Treg cells in this system. However, this is unlikely since continued active Foxp3-driven GFP expression in IL-17A-producing Foxp3^cre^-tdTomato^+^ cells at day 28 argues against this possibility. Moreover, we identified a large population of Th17 that were tdTomato and GFP double negative indicating that transient or persistent Foxp3 expression in Th17 cells does not occur or occurs rarely in our system. If all developing Th17 cells had been actively producing Foxp3 at the time of tamoxifen administration these IL-17A-producing CD4 T cells would have been tdTomato^+^ at days 28 and 48.

While both Foxp3 and RORγt transcription factor are upregulated in the presence of TGF-β, Foxp3 antagonizes RORγt and IL-17A production in the absence of concurrent IL-6 exposure (82–85). The relative ratio of Foxp3 to RORγt within a cell and environmental IL-6 may therefore determine the proinflammatory or regulatory activity of Foxp3^+^ RORγt^+^ cells. Populations of Foxp3^+^ RORγt^+^ cells with Th17 potential have been reported in human peripheral blood (86). These double positive cells were found to have significantly lower expression of Foxp3 than classical suppressive Tregs, although other groups have reported that human pro-inflammatory IL-17A^+^ Foxp3^+^ T cells have significantly higher expression of Foxp3 than classic Tregs (87). Suppressor Foxp3^int.^ RORγt^+^ T cells have been reported in murine autoimmune diabetes but these double positive cells have been reported to produce IL-17 *in vitro* under polarizing conditions (88). Cyclical expression of IL-6 or transient expression of IL-6 by dendritic cells early during persistent exposure to *Pg* may explain the fleeting nature of the Treg plasticity we observed. *In vitro*, purified populations of CD4^+^ CD25^+^ Foxp3^+^ T cells are able to differentiate into Th17 cells in the presence of IL-6 with concurrent absence of exogenous TGF-β (89).

IL-17^+^ Foxp3^+^ T cells have been observed in human chronic inflammatory conditions. For example, IL-17^+^ Foxp3^+^ T cells have been observed in patients with inflammatory bowel disease, or more specifically, in patients with Crohn’s disease but not ulcerative colitis (55, 56). Significantly, IL-17A^+^ Foxp3^+^ T cells are also found in human periodontal lesions but not in gingivitis (30). Lastly, Tregs from human rheumatoid arthritis patients were found to demonstrate increased plasticity toward a Th17-like phenotype (90). While among the studies that evaluated function, IL-17A^+^ Foxp3^+^ T cells were usually found to be suppressive (91, 92), not all studies are in agreement (30, 55, 56, 90). In psoriasis and systemic lupus erythematosus, IL-17^+^ Tregs are pro-inflammatory rather than suppressive (93, 94).

Interestingly, whilst the frequency of IL-17A-expressing Treg cells was up at day 28 and down at day 48, the expression of Foxp3 in the tdTomato^+^ population was stable across both time points. Foxp3*^cre^* tdTomato^+^ T cells remained Foxp3-GFP^+^ and/or stained with anti-Foxp3 mAb, even when producing IL-17A or after 48 days of persistent exposure to *Pg*. This is in agreement with Rubtsov et al. (38) but in contrast to Miyao et al. (95) who found that Foxp3 expression is unstable and transient in conventional CD4^+^ T cells in adoptive transfer models. In this model, expression of IL-17A in cells co-expressing Foxp3 is not explained by transcriptional reprogramming and plasticity of Tregs but rather by transient Foxp3 expression in Th17 cells.

Some of the difference between studies may also be explained by differences in animal model or experimental design. For example, Miyao et al. exposed Foxp3^+^ cells to an inflammatory environment for a period of 4 days *in vitro*. Our model exposed cells to an inflammatory environment *in vivo* for a considerably longer period. It is therefore possible that a longer period of inflammation or more robust inflammatory stimulus is necessary to induce IL-17A production by Foxp3^+^ T cells.

The microbiome likely plays a role in Treg transdifferentiation, as various *Clostridium* species of human origin favor the induction of murine colonic Foxp3^+^ Treg cells co-expressing RORγt (55). At least one study has demonstrated that antibiotic treatment that reduces levels of periodontal pathogens reduces the number of IL-17^+^ Foxp3^+^ T cells from peripheral blood of periodontitis patients, again supporting a connection between inflammatory environment, microbiome, and Treg plasticity (87).

The late-stage response to *Pg*-induced dysbiosis is a switch to a *de novo* Th1-type response sustained by IFN-γ. Although Treg have been shown to produce IFN-γ in several models of disease (36, 96, 97) the frequency of Foxp3^cre^-tdTomato^+^ cells expressing IFN-γ is small (∼2%) and importantly it does not differ between *Pg*-treated and sham-treated mice.

In summary, our data suggests that in a persistent dysbiotic environment driving inflammation the oral CD4^+^ T cell response evolves from one that is initially dominated by IL-17A to one that is predominantly IFN-γ. Such IFN-γ response is generated *de novo* by Th1 cells. Consistent with this shift in the response, we identified a small but significant population of Treg cells expressing IL-17A at day 28 that disappeared at day 48. The kinetics of the inflammatory response may control whether Treg cells will behave as pro- or anti-inflammatory actors. This evolving dysbiosis and inflammatory environment at day 48, post *Pg*, specifically induce Th17 cells into sporadic transdifferentiation and IFN-γ expression. Ultimately, understanding the nature of Treg-Th17 transdifferentiation may provide insights on how to control the inflammatory disease processes. Which components of the microbial biofilm or which host cell under the influence of such microbial environment are responsible for driving the transdifferentiation of Treg or Th17 in the oral environment remains to be elucidated.

## Data Availability Statement

The datasets generated for this study are available on request to the corresponding author.

## Ethics Statement

This study was carried out in accordance with the recommendations of the Association for Assessment and Accreditation of Laboratory Animal Care. The protocol was reviewed and approved by the Institutional Animal Care and Use Committee of the University of Minnesota (Protocol ID #1810-36395A).

## Author Contributions

MC and PB-E provided the intellectual contribution, designed the experiments, and interpreted the data. PB-E, LF, and MC performed the experiments. All authors contributed to drafting the manuscript. PB-E and LF contributed equally to the interpretation of the results.

## Conflict of Interest

The authors declare that the research was conducted in the absence of any commercial or financial relationships that could be construed as a potential conflict of interest.
